# Interferon-Beta Induces Distinct Gene Expression Response Patterns in Human Monocytes versus T cells

**DOI:** 10.1371/journal.pone.0062366

**Published:** 2013-04-23

**Authors:** Noa Henig, Nili Avidan, Ilana Mandel, Elsebeth Staun-Ram, Elizabeta Ginzburg, Tamar Paperna, Ron Y. Pinter, Ariel Miller

**Affiliations:** 1 Division of Neuroimmunology and Multiple Sclerosis Center, Carmel Medical Center, Haifa, Israel; 2 Department of Computer Science, Technion-Israel Institute of Technology, Haifa, Israel; 3 Rappaport Faculty of Medicine & Research Institute, Technion-Israel Institute of Technology, Haifa, Israel; University of Cape Town, South Africa

## Abstract

**Background:**

Monocytes, which are key players in innate immunity, are outnumbered by neutrophils and lymphocytes among peripheral white blood cells. The cytokine interferon-β (IFN-β) is widely used as an immunomodulatory drug for multiple sclerosis and its functional pathways in peripheral blood mononuclear cells (PBMCs) have been previously described. The aim of the present study was to identify novel, cell-specific IFN-β functions and pathways in tumor necrosis factor (TNF)-α-activated monocytes that may have been missed in studies using PBMCs.

**Methodology/Principal Findings:**

Whole genome gene expression profiles of human monocytes and T cells were compared following *in vitro* priming to TNF-α and overnight exposure to IFN-β. Statistical analyses of the gene expression data revealed a cell-type-specific change of 699 transcripts, 667 monocyte-specific transcripts, 21 T cell-specific transcripts and 11 transcripts with either a difference in the response direction or a difference in the magnitude of response. RT-PCR revealed a set of differentially expressed genes (DEGs), exhibiting responses to IFN-β that are modulated by TNF-α in monocytes, such as *RIPK2* and *CD83*, but not in T cells or PBMCs. Known IFN-β promoter response elements, such as ISRE, were enriched in T cell DEGs but not in monocyte DEGs. The overall directionality of the gene expression regulation by IFN-β was different in T cells and monocytes, with up-regulation more prevalent in T cells, and a similar extent of up and down-regulation recorded in monocytes.

**Conclusions:**

By focusing on the response of distinct cell types and by evaluating the combined effects of two cytokines with pro and anti-inflammatory activities, we were able to present two new findings First, new IFN-β response pathways and genes, some of which were monocytes specific; second, a cell-specific modulation of the IFN-β response transcriptome by TNF-α.

## Introduction

T cells comprise 25–30% and monocytes constitute 3–10% of all circulating leukocytes in a normal individual [1]. The distinct activities of T cell subsets, monocytes, and monocyte-derived dendritic cells and macrophages are determined in part by the cytokines they secrete into the environment, such as TNF-α, IFN-β and others [2]–[5]. Monocytes are part of the innate immune system, which is the first line of host defense against infections. Activated monocytes can function as antigen-presenting cells and as immune effector cells [6]–[8]. Aberrations of monocyte function are increasingly recognized as important in different autoimmune diseases, such as systemic lupus erythematosus and multiple sclerosis (MS) [9], [10]. As a result, monocytes are becoming an appealing target for therapeutic strategies which are aimed at manipulating monocyte-mediated immune responses [11], [12].

Type I interferons (IFNs) are cytokines that are secreted in response to viral and bacterial infections, and are involved in the elicitation of antiviral, antiproliferative, and immunomodulatory responses [13]. IFN-α and IFN-β induce their biological effects by binding to a heterodimeric cell surface receptor complex (IFNAR) which is composed of two distinct transmembrane proteins, namely IFNAR1 and IFNAR2. This interaction activates the Janus-activated kinase (JAK)/signal transducer and activator of transcription (STAT) signaling pathway, and results in the assembly of IFN-stimulated gene factor 3 (ISGF3) complexes and STAT1 homodimers. These complexes then translocate into the cell nucleus where they activate the transcription of many different genes by binding to IFN-stimulated response elements (ISREs) or IFN-γ-activating sites (GAS) in their promoters. Ligand binding to IFNAR can also lead to activation of mitogen-activated protein kinase (MAPK) signaling cascades, including the p38 and the phosphatidylinositol 3-kinase cascades [14], [15].

Recombinant IFN-α and IFN-β have been approved for the treatment of numerous diseases, which include hepatitis B and C, melanoma, T cell lymphomas, and MS [16]. In fact, IFN-β was the first drug to be approved for use in MS patients, and is still one of the most widely used drugs for treating patients with relapsing MS [17]. Since up to 50% of all MS patients do not benefit from IFN-β treatment or respond only partially, genetic biomarkers that are predictive of the patient’s response to IFN-β therapy are of major importance, and are the focus of many studies [17]–[19]. To this end, some investigators analyzed the whole genome transcription profiles of peripheral blood mononuclear cells (PBMCs), which are a mixture of several leukocyte subsets [20]–[22]. Other investigators have analyzed the expression of a few selected genes, such as *FOXP3* or *IFNAR2*, in specific cell subsets, such as regulatory T cells [23], [24]. None of the studies conducted to date have been successful in identifying clinically relevant genetic biomarkers for response to therapy [25].

The study of Van Boxel-Dezaire et al. highlighted the differential effects of IFN-β on apoptosis-related genes in various leukocyte subsets [26]. Specifically, these researchers found that IFN-β up-regulated apoptosis-inducing genes in monocytes, but not in T cells, and that this up-regulation was associated with the activation of the STAT1 protein. In a different study, Waddell et al. reported on cell-specific changes in gene expression profiles after IFN-γ induction [27]. Together, these studies emphasize the importance of studying gene expression profiles in separated leukocyte populations.

Blood monocytes may encounter IFN-β during infections, or when administered as a long-term immunomodulatory therapy in MS. One of the cytokines elevated in these states is the pro-inflammatory TNF-α [28], [29]. Only a few *in vitro* studies have considered the effects of TNF-α on the cellular response to IFN-β; however, from these studies it is clear this cross-interaction is complex and cell type-dependent [3]. We hypothesized that IFN-β elicits a cell-specific gene expression response in monocytes, which may be modulated by the pro-inflammatory cytokines in the extracellular milieu under conditions of infection or inflammatory disease. Furthermore, we surmised that the cell-specific response of monocytes to cytokines might have been obscured by the response of the more abundant cells in the PBMC population, such as T cells. Accordingly, the aim of the present study was to dissect the transcriptional profile of TNF-α-activated monocytes following exposure to IFN-β, using networks and pathways analysis tools.

## Results

### Analysis of Microarray Data Shows Distinct IFN-β Gene Expression Response Profiles in Monocytes and T Cells

**Figure 1:**
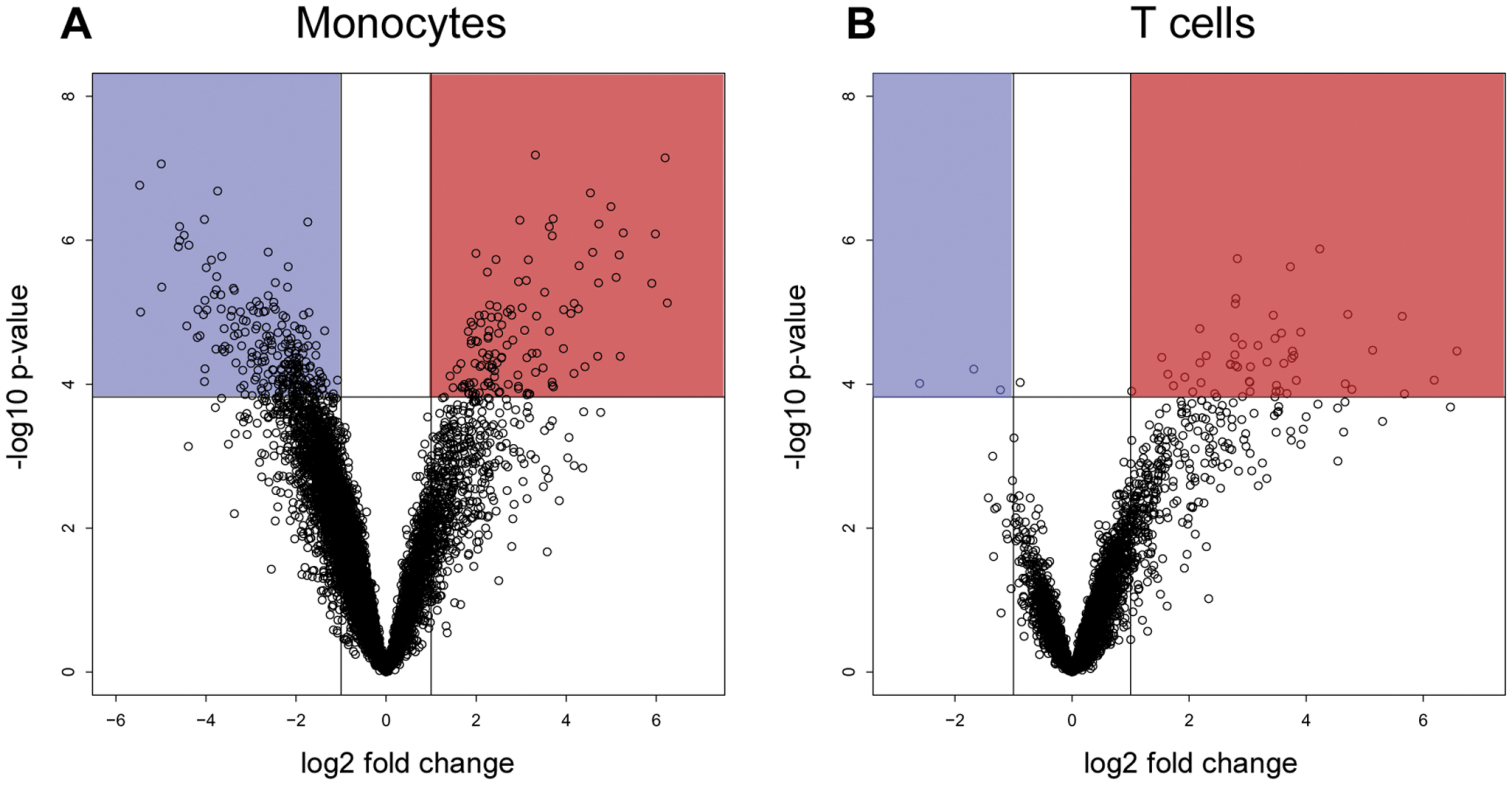
Volcano plots for the differential gene expression following IFN-β treatment of monocytes and T cells. A. monocytes; B. T cells. The X axis describes the fold change in expression levels between cells treated with IFN-β relative to untreated cells, for each transcript in a log2 scale. The Y axis shows the statistical significance expressed as -log10(p-value) from the simple comparison. Transcripts with log2 difference of ≥1 and with -log10(p-value) ≥3.8, which is the equivalent of p≤0.05 after FDR adjustment, were defined as differentially expressed genes (DEGs) and are highlighted with blue for down-regulated and red for up-regulated DEGs.

**Figure 2:**
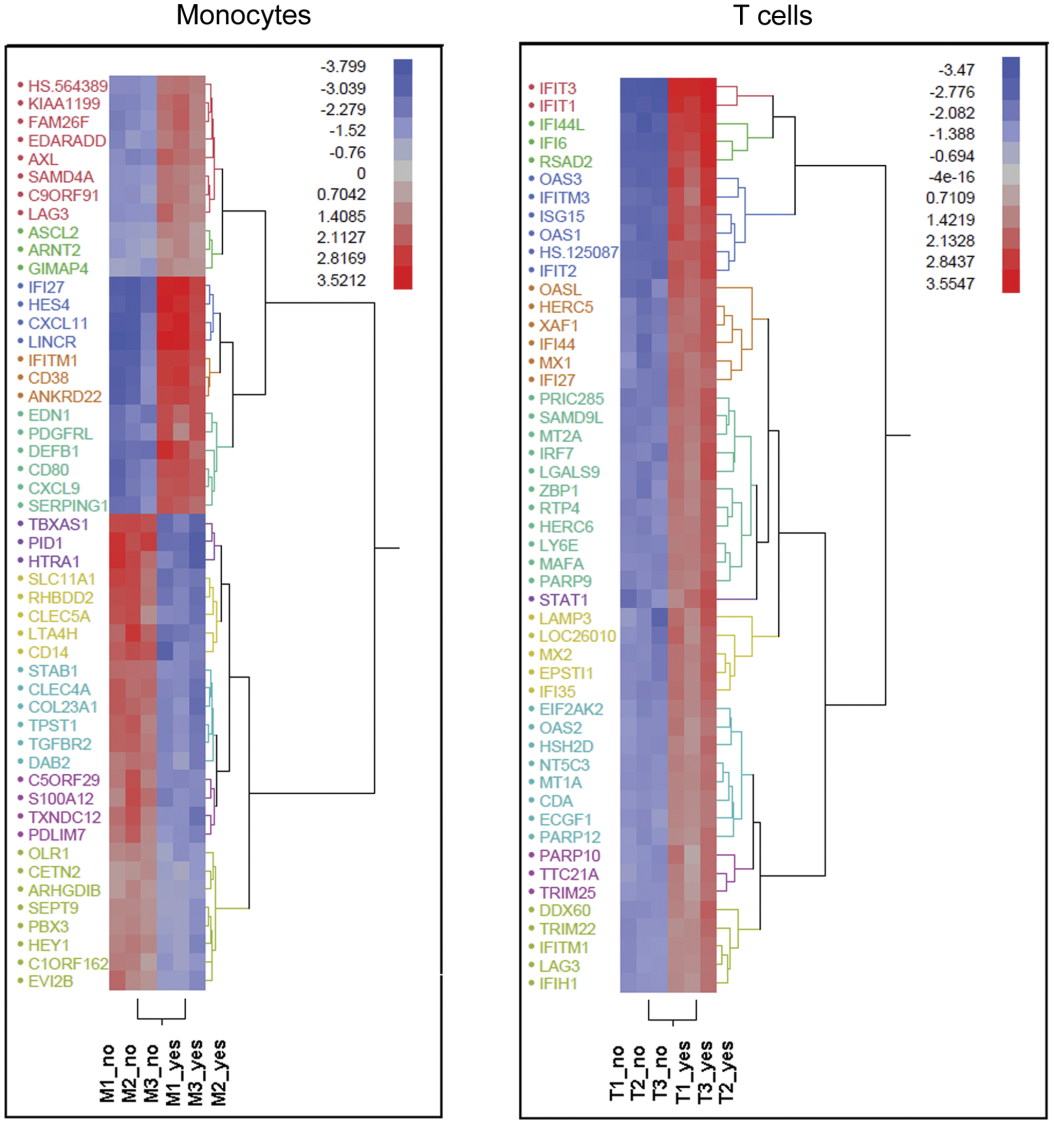
Cluster analysis of DEGs in monocytes and in T lymphocytes. Hierarchical clustering of the 50 most DEGs for IFN-β treatment in monocytes and T cells as sorted by fold change [P(IFNβ)≤0.05 within each cell type]. Expression values (in log2 scale) are color coded from low expression in blue to high expression in red. The first three columns from the left show untreated samples (marked as 'no') and the next 3 are IFN-β-treated cells (marked as 'yes'). Genes that have a similar expression level have a common gene symbol color.

We compared the gene expression profiles of human TNF-α activated monocytes and T cells following exposure to IFN-β, using Illumina’s BeadArray™ microarray technology. The pre-activation with TNF-α was done in order to simulate a pro-inflammatory state in the cells at the time of exposure to IFN-β. The study workflow is shown in Fig. S1. Analysis of the IFN-β effect within each cell type revealed the presence of 2113 and 242 differentially expressed genes (DEGs) (≥ twofold change at adjusted p-value of 0.05) in monocytes and T cells respectively, with 106 transcripts common to both cell types (Table 1). In addition, following IFN-β exposure a cell-type specific change of 699 transcripts was revealed with 667 monocyte-specific transcripts, 21 T cell-specific transcripts (Tables 2 and 3), and 11 transcripts with either a difference in the response direction, for example RARA, or a difference in the magnitude of response, for example CD38. The T cell IFN-β response appeared to involve a smaller number of genes compared to the monocyte response (Fig. 1). Moreover, the overall directionality of the gene expression regulation by IFN-β was different in T cells and monocytes, with up-regulation more prevalent in T cells, and a similar extent of up and down-regulation recorded in monocytes (Figs. 1 and 2). The hierarchical clustering displayed in figure 2 presents the 50 top DEGs in each cell type, ranked according to the highest difference in expression. This figure highlights the small variability in expression levels across the biological replicates within cell type.

**Table 1:** Differentially expressed genes in both monocytes and T cells (25 out of 106 genes).

Gene	DEFINITION	Monocytes		T cells		Cell-type*IFN-β
		Fold change^1^	P-value^2^	Fold change^1^	P-value^2^	P-value^3^
LAG3	Lymphocyte-activation gene 3 (LAG3)	8.66	2e−4	6.91	4e−4	0.48
TLR7	Toll-like receptor 7 (TLR7)	6.92	5e−4	2.85	0.006	0.06
NT5C3	5′-nucleotidase, cytosolic III (NT5C3)	10.1	5e−4	7.51	0.001	0.47
SLC38A5	Solute carrier family 38, member 5 (SLC38A5)	4.89	5e−4	2.4	0.006	0.07
ISG20	Interferon stimulated exonuclease gene 20kDa (ISG20)	15.3	0.001	5.3	0.007	0.11
DUSP5	Dual specificity phosphatase 5 (DUSP5)	10.2	0.001	2.63	0.033	0.06
ZBP1	Z-DNA binding protein 1 (ZBP1)	9.45	0.001	11.9	0.001	0.63
LGALS3BP	Lectin, galactoside-binding, soluble, 3 binding protein (LGALS3BP)	9.13	0.001	5.1	0.004	0.25
CD14	CD14 molecule (CD14)	0.11	0.001	0.38	0.034	0.06
GIMAP6	GTPase, IMAP family member 6 (GIMAP6)	5.32	0.001	2.06	0.039	0.07
HS.561105	BX116726 NCI_CGAP_Pr28 cDNA clone IMAGp998J065569 sequence	4.78	0.002	2.75	0.011	0.18
JAK2	Janus kinase 2 (a protein tyrosine kinase) (JAK2)	3.34	0.002	2.82	0.004	0.57
HSH2D	Hematopoietic SH2 domain containing (HSH2D)	7.67	0.002	8.21	0.002	0.9
IFITM2	Interferon induced transmembrane protein 2 (1–8D) (IFITM2)	7.8	0.002	4.91	0.007	0.39
CBX7	Chromobox homolog 7 (CBX7)	0.18	0.002	0.46	0.049	0.09
HS.537991	HUMGS0004661 Human adult (K.Okubo) cDNA 3 sequence	5.47	0.003	2.93	0.016	0.19
AIM2	Absent in melanoma 2 (AIM2)	13.6	0.003	6.67	0.01	0.32
DYNLT1	Dynein, light chain, Tctex-type 1 (DYNLT1)	4.01	0.003	3.26	0.005	0.58
LOC728216	PREDICTED: similar to ubiquitin specific peptidase 18 (LOC728216)	4.92	0.003	3.74	0.006	0.52
SP140	SP140 nuclear body protein (SP140)	3.98	0.003	3.66	0.004	0.83
ATF3	Activating transcription factor 3 (ATF3)	4.94	0.003	2.17	0.048	0.11
MOV10	Mov10, Moloney leukemia virus 10, homolog (mouse) (MOV10)	3.64	0.003	3.67	0.003	0.98
GBP4	Guanylate binding protein 4 (GBP4)	9.43	0.003	4.28	0.019	0.23
TNFSF10	Tumor necrosis factor (ligand) superfamily, member 10 (TNFSF10)	27.2	0.003	10	0.015	0.3
GBP5	Guanylate binding protein 5 (GBP5)	8.77	0.003	6.13	0.007	0.57

1 Expression level in treated samples divided by expression level in untreated samples.

2 FDR adjusted p-value for IFN-β effect within each cell type.

3 FDR adjusted p-value of the two-way ANOVA for the cell-type*IFN-β interaction.

**Table 2:** Differentially expressed genes in monocytes but not in T cells (25 out of 667).

Gene	DEFINITION	Monocytes		T-cells		Cell-type*IFN-β
		Fold change^1^	P-value^2^	Fold change^1^	P-value^2^	P-value^3^
HS.564389	DKFZp686C15231_r1 686 (synonym: hlcc3)	9.95	3.6e−5	1.14	0.4	0.003
AXL	AXL receptor tyrosine kinase (AXL)	13.1	3.9e−5	1.25	0.22	0.003
STAB1	stabilin 1 (STAB1)	0.07	3.9e−5	1.11	0.6	0.003
TBXAS1	Thromboxane A synthase 1 (platelet, cytochrome P450, family 5, subfamily A) (TBXAS1)	0.03	4e−5	0.84	0.5	0.003
PID1	Phosphotyrosine interaction domain containing 1 (PID1)	0.02	5e−5	1.02	0.96	0.003
DEFB1	Defensin, beta 1 (DEFB1)	31.8	0.0001	1.18	0.58	0.004
EDN1	endothelin 1 (EDN1)	23.2	0.0001	1.69	0.06	0.005
SAMD4A	Sterile alpha motif domain containing 4A (SAMD4A)	7.84	0.0001	1.47	0.04	0.005
CLEC4A	C-type lectin domain family 4, member A (CLEC4A)	0.06	0.0001	1.05	0.86	0.004
QSOX1	Quiescin Q6 sulfhydryl oxidase 1 (QSOX1)	0.3	0.0001	0.93	0.51	0.005
KIAA1199	KIAA1199 (KIAA1199)	12.3	0.0001	1.18	0.49	0.005
SLC11A1	Solute carrier family 11 (proton-coupled divalent metal ion transporters),member 1 (SLC11A1)	0.04	0.0001	1.04	0.92	0.005
OLR1	Oxidized low density lipoprotein (lectin-like) receptor 1 (OLR1)	0.16	0.0001	1.04	0.84	0.005
CD80	CD80 molecule (CD80)	26.9	0.0001	1.6	0.16	0.008
FOS	v-fos FBJ murine osteosarcoma viral oncogene homolog (FOS)	0.06	0.0001	1.04	0.92	0.005
LTA4H	Leukotriene A4 hydrolase (LTA4H)	0.04	0.0001	0.74	0.35	0.007
RHBDD2	Rhomboid domain containing 2 (RHBDD2)	0.05	0.0001	0.99	0.98	0.005
HTRA1	HtrA serine peptidase 1 (HTRA1)	0.03	0.0001	1.07	0.88	0.005
CXCL9	Chemokine (C-X-C motif) ligand 9 (CXCL9)	24.1	0.0001	1.79	0.1	0.01
C5ORF29	Chromosome 5 open reading frame 29 (C5orf29)	0.08	0.0002	0.85	0.58	0.008
SEPT9	Septin 9 (SEPT9)	0.18	0.0002	0.81	0.27	0.01
ASCL2	Achaete-scute complex homolog 2 (Drosophila) (ASCL2)	5.43	0.0002	1.85	0.01	0.018
TPST1	Tyrosylprotein sulfotransferase 1 (TPST1)	0.07	0.0002	0.94	0.87	0.008
COL23A1	Collagen, type XXIII, alpha 1 (COL23A1)	0.06	0.0002	1.17	0.63	0.006
SERPING1	Serpin peptidase inhibitor, clade G (C1 inhibitor), member 1 (angioedema,hereditary) (SERPING1)	19.5	0.0002	1.3	0.44	0.01

1 Expression level in treated samples divided by expression level in untreated samples.

2 FDR adjusted p-value for IFN-β effect within each cell type.

3 FDR adjusted p-value of the two-way ANOVA for the cell-type*IFN-β interaction.

**Table 3:** Differentially expressed genes in T cells but not in monocytes (21 in total).

Gene	DEFINITION	Monocytes		T-cells		Cell-type*IFN-β
		Fold change^1^	P-value^2^	Fold change^1^	P-value^2^	P-value^3^
AZI2	5-azacytidine induced 2 (AZI2)	0.65	0.139	2.23	0.018	0.036
C1ORF173	Chromosome 1 open reading frame 173 (C1orf173)	1.31	0.207	2.95	0.002	0.045
CD68	CD68 antigen (CD68)	0.9	0.792	4	0.005	0.037
CDA	Eytidine deaminase (CDA)	1.998	0.011	7.08	2e−4	0.016
CMTM8	CKLF-like MARVEL transmembrane domain containing 8 (CMTM8)	0.58	0.104	2.11	0.041	0.043
CYP2J2	Cytochrome P450, family 2, subfamily J, polypeptide 2 (CYP2J2)	1.04	0.933	6.52	0.001	0.022
ECGF1	Endothelial cell growth factor 1 (platelet-derived) (ECGF1)	0.93	0.8	6.96	3e−4	0.01
GORASP1	Golgi reassembly stacking protein 1, 65kDa (GORASP1)	0.82	0.58	2.77	0.014	0.049
HS.386275	cl02h05.z1 Hembase; Erythroid Precursor Cells (LCB:cl library)	0.99	0.991	3.79	0.007	0.048
HS.570308	cDNA clone IMAGp998F201004 sequence	0.9	0.752	2.58	0.011	0.049
LPIN2	Lipin 2 (LPIN2)	0.68	0.207	2.59	0.013	0.035
MKRN1	Makorin, ring finger protein, 1 (MKRN1)	0.82	0.42	2.09	0.013	0.043
PIM2	Pim-2 oncogene (PIM2)	0.62	0.239	2.71	0.029	0.047
PRKD2	Protein kinase D2 (PRKD2)	0.83	0.545	4.18	0.002	0.021
RASGRP3	RAS guanyl releasing protein 3 (calcium and DAG-regulated) (RASGRP3)	1.99	0.024	5.14	0.001	0.048
RGS1	Regulator of G-protein signaling 1 (RGS1)	1.22	0.421	3.32	0.002	0.038
SLFN13	Schlafen family member 13 (SLFN13)	0.84	0.654	4.33	0.004	0.03
SOBP	Sine oculis binding protein homolog (Drosophila) (SOBP)	0.82	0.455	3.06	0.003	0.023
TRIM25	Tripartite motif-containing 25 (TRIM25)	1.52	0.18	6.85	0.001	0.028
TTC21A	Tetratricopeptide repeat domain 21A (TTC21A)	1.34	0.386	6.88	0.001	0.028
FCHSD2	Delta-like 1 (Drosophila) (DLL1)	0.48	0.073	2.37	0.044	0.04

1 Expression level in treated samples divided by expression level in untreated samples.

2 FDR adjusted p-value for IFN-β effect within each cell type.

3 FDR adjusted p-value of the two-way ANOVA for the cell-type*IFN-β interaction.

### Validation of the Gene Expression Microarray Results by Quantitative Real-time PCR

**Figure 3:**
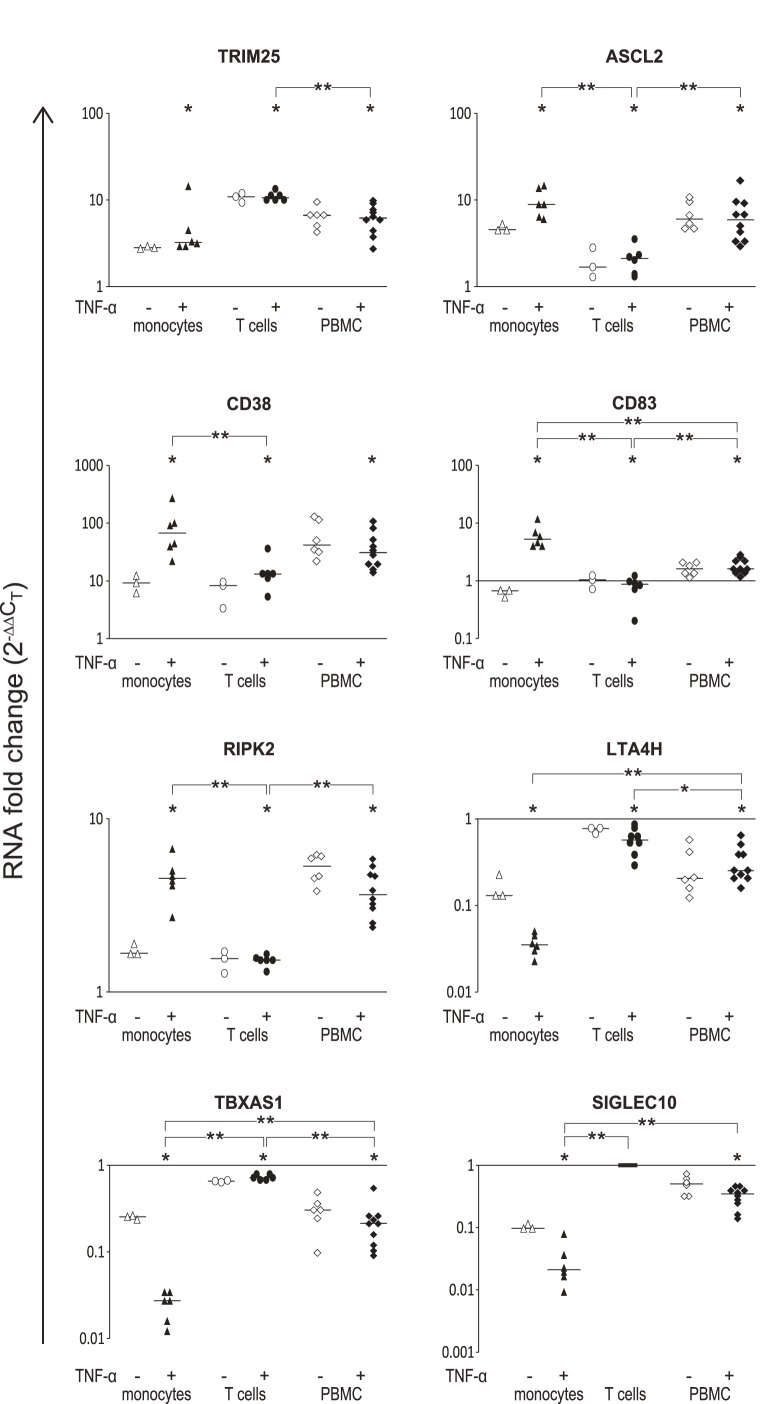
RT-PCR confirms differential expression between monocytes and T cells in response to IFN-β. Monocytes, T cells, and PBMCs from healthy donors were included in the validation of eight DEGs selected from the microarray data. Triangles represent monocytes, circles- T cells, and diamonds- PBMCs. Full and empty symbols represent cells pre-incubated or not with TNF-α. The horizontal bars mark the median values. The Y axis depicts the changes in expression levels in response to IFN-β as fold change (2^−ΔΔCT^); *p-values<0.03 for the IFN-β response (Wilcoxon signed rank test). For difference in fold change between monocytes, T cells, and PBMCs, *p-value<0.05; **p-value <0.01 (Mann Whitney test). The mRNA levels of *SIGLEC10* were below detection level by RT-PCR in T cells, under all conditions, therefore the p-value for comparison between monocytes and T cells was calculated under the assumption of no change in expression (fold change = 1, marked by rectangle) in T cells.

Seven genes from the monocytes-specific DEG list were selected for further validation by real time (RT)-PCR analysis at the same TNF-α and IFN-β exposures used for the gene array experiment. The selection criteria included, in addition to a significant P value for interaction (cell-type*IFN-β), lack of previous reports at the time of analysis as an IFN-β response gene, and functional relevance to monocyte activity. The seven selected genes encode: (a) *CD38*, a multifunctional ectoenzyme that is involved in cell adhesion, signal transduction, and calcium signaling [30]; (b) *CD83,* known as a marker for dendritic cell activation, that is involved in CD4^+^ T cell maturation and B cell receptor signaling [4], [31], [32]; (c) *ASCL2*, a transcription factor [33]; (d) *LTA4H*, a bifunctional zinc metalloenzyme [34]; (e) *RIPK2,* a kinase which has been associated with apoptosis induction and implicated in Nod1 and Nod2 signaling [35], [36]; (f) *SIGLEC10*, a sialic acid binding cell surface protein involved in regulation of circulating levels of inflammatory cytokines [37]; and (g) *TBXAS1,* encoding the synthase for thromboxane A, which promotes platelet aggregation and is a potent vasoconstrictor [38]. In addition, *TRIM25/EFP*, which is known to be up-regulated by IFN-β [39] and was detected as a DEG in T cells in this study, was used as a positive control for IFN-β effect. Analysis of the IFN-β response following pre-exposure to TNF-α for these genes was performed in parallel in PBMCs, to assess the cytokine effects in a mixed cell population that include cell-cell interactions and an averaging effect of the combined activities of the different cell types. The change in expression levels following IFN-β treatment was significant for all eight genes in the monocytes, T cells, and PBMCs, except for *CD83* and *SIGLEC10* in T cells (Fig. 3). A significant difference in response was observed for all genes between the monocytes and T cells, whereas the PBMC response was intermediate between the levels of transcripts in the monocytes and in the T cells (Fig. 3).

### The TNF-α-activated Monocytes Display an Enhanced Differential Transcriptional Response to IFN-β for Selected Genes

In order to evaluate the contribution of TNF-α to the monocytes' overall response to IFN-β, we repeated the RT-PCR analysis of the IFN-β response in monocytes that had not been pretreated by TNF-α (Fig. 3). Pre-incubation of the monocytes with TNF-α increased the magnitude of the IFN-β response for seven of the tested genes. Interestingly, up- or down-regulation of *CD83* expression in monocytes by IFN-β depended on whether the monocytes were pre-exposed to TNF-α or not. In contrast, pre-incubation with TNF-α did not affect the IFN-β response in T cells and in PBMCs. These results suggest that a prior TNF-α exposure of leukocytes can modulate the IFN-β response in a cell-specific manner.

### IFN-β Increases CD38 Protein Expression in Monocytes but not in T Cells

**Figure 4:**
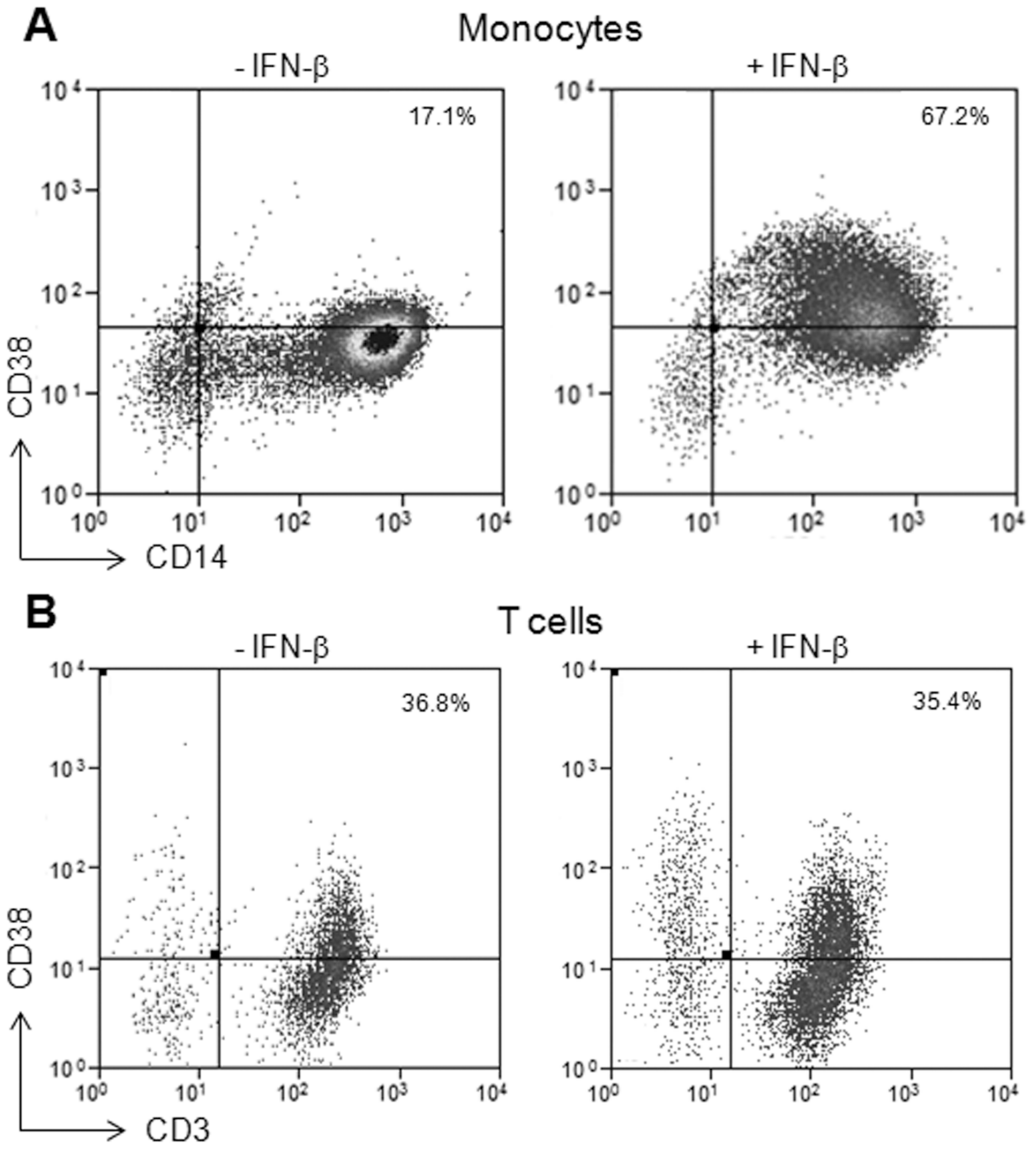
CD38 protein expression is increased in monocytes, but not in T cells, in response to IFN-β. Flow cytometry analysis of CD38 expression in monocytes and T cells incubated with TNF-α for 2 hours and then treated with IFN-β for 40 hours. Representative results of CD38 expression with and without IFN-β treatment in A. CD14+ monocytes and B. CD3+ T cells. N≥3 for each cell type.

To determine whether the IFN-β response seen at the RNA level also extends to the encoded protein level, we measured CD38 protein levels by flow cytometry. We did not detect any change in CD38 protein expression in either monocytes or T cells after 16 hours of IFN-β exposure; however, following a longer exposure of 40 hours to IFN-β a significant increase in CD38 expression was observed, and in monocytes only (Fig. 4). Thus, the protein analysis for CD38 in monocytes corroborates the differential cell-specific results obtained at the RNA level.

### Enriched Biological Functions within the IFN-β Response DEGs

Four bioinformatics tools were used to identify enriched biological functions and gain insight on possible functional consequences of the changes in gene expression elicited by IFN-β: (a) GOrilla, a program that identifies enriched Gene Ontology (GO) terms in ranked lists of transcripts [40], (b) SPIKE, a program for detecting enriched signaling pathways, (c) PRIMA, a program for identifying transcription factor binding sites which are enriched in gene promoters [41], [42], and (d) Ingenuity Pathway Analysis (IPA) (Ingenuity® Systems, www.ingenuity.com), a program for detecting known canonical pathways that are enriched with the DEGs and for construction of new networks of interactions between the DEGs.

**Figure 5:**
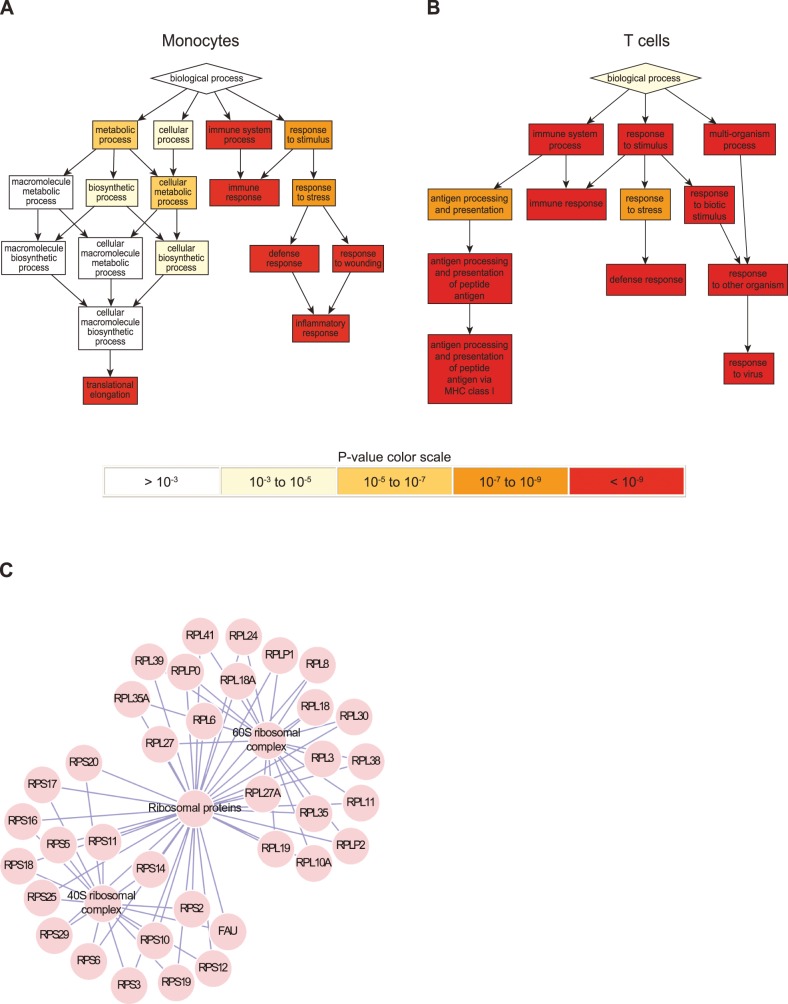
Biological processes induced by IFN-β in monocytes and T cells. The differentially expressed transcript lists were ranked by the simple comparison p-value and fold change, and then evaluated for functional enrichment by GOrilla. Visual representations of the enriched gene ontology terms produced by GOrilla are shown for A. monocytes and B. T cells. The significance of the enrichment of the biological processes is color coded as indicated. C. Output produced by SPIKE [42] showing enrichment of the 'Translation elongation' pathway in monocytes (p = 10^−65^).

The complete gene lists, ranked by the p-value and fold change within each cell type, were uploaded into GOrilla. Almost all of the top enriched GO terms that were identified by GOrilla were directly related to immune system activity in both cell types, which is in agreement with the expected effects of IFN-β (Fig. 5A and Table 4). However, despite the large number of IFN-β response DEGs identified in monocytes GOrilla did not identify any novel functional network connecting these DEGs. We deduced that the paucity of functional links between the monocyte DEGs may result from the poor representation of biological interactions data for these genes in the available databases.

**Table 4:** Enriched biological functions GO terms found in ranked gene lists^1^.

	GO Term	Description	p-value	Enrichment^2^
Monocytes	GO:0006952	defense response	3E−16	2.7
	GO:0002376	immune system process	9E−14	2.15
	GO:0009611	response to wounding	2E−13	3.24
	GO:0006954	inflammatory response	1E−12	3.31
	GO:0006955	immune response	2E−10	2.22
	GO:0006414	translation elongation	4E−10	1.97
T cells	GO:0009615	response to virus	3E−23	22.06
	GO:0002376	immune system process	1E−22	3.34
	GO:0006955	immune response	4E−19	3.9
	GO:0051707	response to other organism	9E−19	14.67
	GO:0009607	response to biotic stimulus	2E−17	6.57
	GO:0051704	multi-organism process	4E−14	4.62
	GO:0050896	response to stimulus	1E−12	1.79
	GO:0002474	antigen processing and presentation of peptide antigen viaMHC class I	1E−11	6.17
	GO:0006952	defense response	2E−11	2.95
	GO:0048002	antigen processing and presentation of peptide antigen	3E−10	5.05

1 The data in this table was generated by GOrilla [40]. Input lists contained all transcripts expressed in T cells and monocytes sorted by p-value and then fold change. GO terms presented have a p-value ≤2E−10,

2 The ‘enrichment’ value is the ratio between the number of genes associated with a specific GO term within a target set and the total number of genes associated with that GO term.

The most prominent pathway that was identified in the monocyte DEGs by SPIKE was ’translation elongation‚ (116-fold enrichment with a p-value of 10^−65^). This pathway, which has many ribosomal subunit proteins involved in protein translation, was found to be down-regulated by IFN-β (Fig. 5B). SPIKE did not identify any significantly enriched pathways from the list of T cell DEGs.

We next used the PRIMA module to identify enrichment of specific promoter motifs within the gene lists. DEG lists for each cell type were uploaded either as cell-specific lists, after excluding the common T cell and monocyte DEGs set, or as a complete list of DEGs, for each cell type. Neither ISRE, nor any other transcription factor binding site motif, was enriched in any of the monocytes DEG lists. In contrast, the ISRE, IFN regulatory factor (IRF)-7, and IRF-1 motifs were identified in both the T cell-specific list and in the T cell complete DEGs list. To account for a possible effect of the large discrepancy in the monocytes and T cells DEG list sizes on the analysis, we repeated PRIMA analysis on equal-sized lists of top 100 and 200 DEGs for each cell type. Whereas ISRE was enriched in the T cell lists (in 33 DEGs out of 100, P = 10^−14^, and in 74 DEGs out of 200, P = 10^−26^), no known transcription factor binding site motif was enriched in the monocyte DEGs lists.

**Figure 6:**
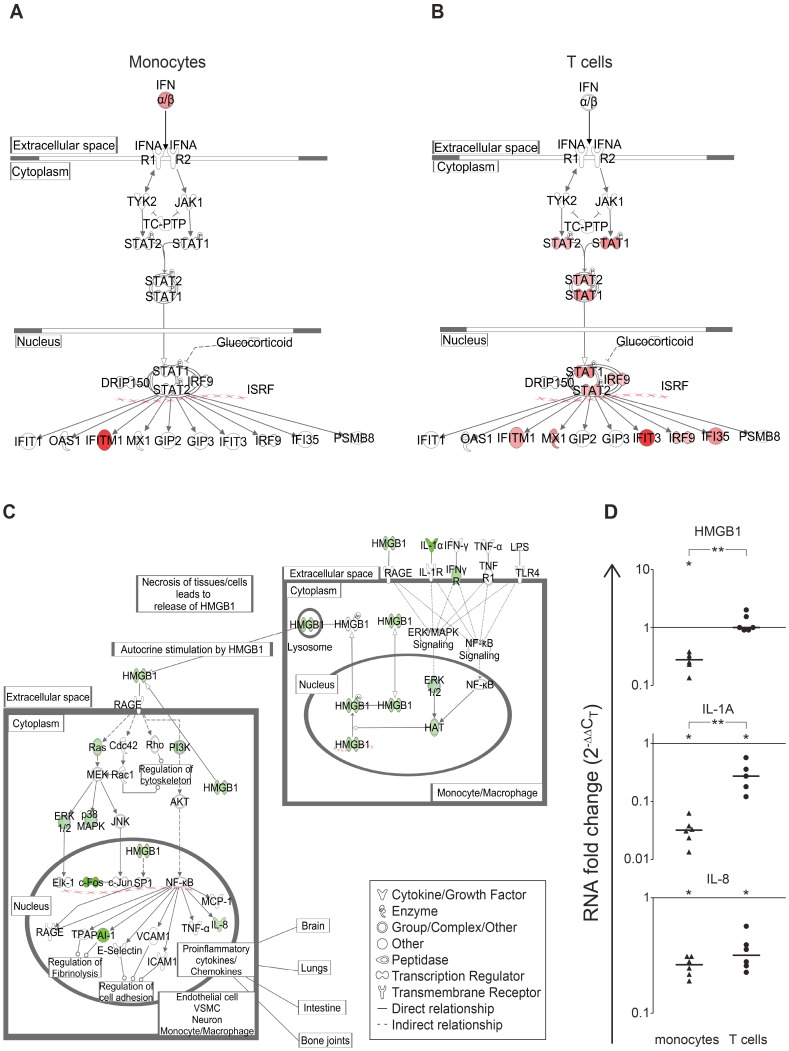
Canonical pathways involved in IFN-β-mediated modulation in monocytes and T cells. DEGs following IFN-β treatment were mapped to canonical pathways using IPA. A. monocytes (p = 1.4*10^−2^); B. T cells (p = 1.4*10^−14^); C. DEGs from monocytes were enriched in the canonical HMGB1 signaling pathway (p = 3.37*10^−4^). VSMC- vascular smooth endothelial cell. The HMGB1 pathway was not enriched in T cell DEGs. Green indicates down-regulation and red indicates up-regulation, with lower p-values represented by more intense colors. The known functions of the proteins included in the pathways are denoted in the symbol legend box. D. RT-PCR analyses for the genes *HMGB1*, *IL1A* and *IL8* from the HMGB1 signaling pathway for monocytes and T cells (n = 6). The Y axis depicts the changes in RNA expression levels in response to IFN-β as fold change (2^−ΔΔCT^), *p-value<0.05 for the IFN-β response (Wilcoxon signed rank test). For the difference in fold change between monocytes and T cells, **p-value ≤0.01 (Mann Whitney test). Horizontal bars indicate median values for each column of data points.

IPA ranked canonical immune-related pathways among the top pathways incorporating the DEGs detected in the IFN-β-treated monocytes and T cells (Table 5). Notably, both the IFN response and the interferon regulatory factor (IRF) canonical pathways were ranked among the top ten pathways for T cells, but not for monocytes. The canonical IFN response pathway in the T cells (Figs. 6A and 6B) was highly enriched with DEGs when compared to that of the monocytes. One of the novel pathways that was enriched with DEGs in the IFN-β-treated monocytes was the ’high-mobility group box-1 (HMGB1) signaling pathway’, a cytokine pathway which is known to be activated in inflammatory conditions [43] and has not yet been reported as part of the IFN-β response (Table 5). Several members of the HMGB1 pathway were down-regulated following IFN-β treatment of monocytes (Fig. 6C). RT-PCR analysis corroborated that IFN-β down-regulates in monocytes the gene expression of *HMGB1*, *IL1A*, and *IL8* (Fig. 6C). The IFN-β response for *HMGB1* and *IL1A* in the monocytes was significantly different from that of the T cells (Fig. 6D).

**Table 5:** Ten top canonical pathways enriched in monocytes and T cells datasets.

	Canonical pathway	p-value	Ratio^1^
Monocytes	Communication between Innate and Adaptive Immune Cells	2E−5	0.12
	Role of Pattern Recognition Receptors in Recognition of Bacteria and Viruses	1E−4	0.14
	TREM1 Signaling	1E−4	0.15
	Leukocyte Extravasation Signaling	3E−4	0.10
	HMGB1 Signaling	3E−4	0.13
	Systemic Lupus Erythematosus Signaling	3E−4	0.09
	IL-6 Signaling	1E−3	0.12
	Chemokine Signaling	1E−3	0.13
	T Cell Receptor Signaling	1E−3	0.11
	IL-3 Signaling	2E−3	0.13
T cells	Interferon Signaling	1E−14	0.28
	Activation of IRF by Cytosolic Pattern Recognition Receptors	5E−10	0.12
	Role of Pattern Recognition Receptors in Recognition of Bacteria and Viruses	5E−8	0.09
	Retinoic acid Mediated Apoptosis Signaling	6E−5	0.07
	Role of RIG1-like Receptors in Antiviral Innate Immunity	2E−4	0.08
	TREM1 Signaling	6E−4	0.06
	JAK/Stat Signaling	1E−2	0.04
	PDGF Signaling	1E−2	0.04
	IL-15 Production	2E−2	0.06
	Pyrimidine Metabolism	2E−2	0.02

1 Ratio indicates the number of DEGs participating in a pathway divided by the total number of molecules participating in that pathway. Data in this table was generated by IPA.

**Figure 7:**
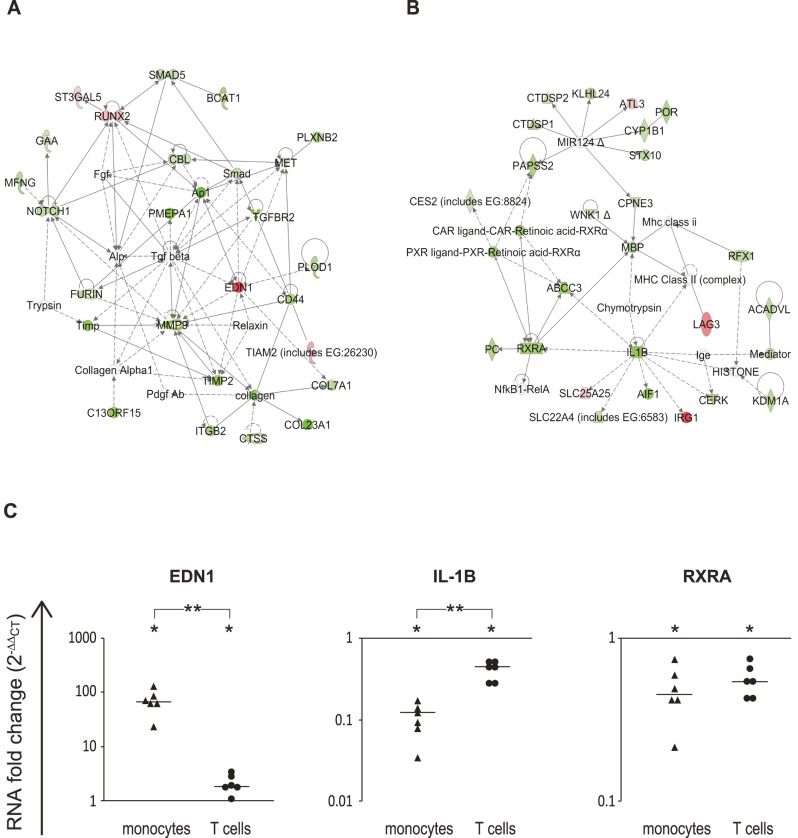
Networks regulated by IFN-β in monocytes include functions of cell migration, and cellular development and proliferation. Genes were mapped to IPA-generated networks based on known gene interactions, and the networks were ranked according to the number of biological connections between the transcripts by IPA. Shown in this figure are two high-score networks (IPA score = 29 for both), which we named based on the IPA-suggested keywords: A. Cell migration; B. Cellular development and proliferation. Green nodes indicate down-regulation and red indicate up-regulation. White nodes indicate molecules that were incorporated into the network through relationships with other molecules but are not DEGs. Dotted lines represent indirect interaction, solid lines represent direct interaction. Protein symbols are explained in the symbol legend in figure 6C. C. RT-PCR analyses for the genes *EDN1* from Cell migration network, and *IL1B* and *RXRA* from cellular development and proliferation network for monocytes and T cells (n = 6). The Y axis depicts the changes in RNA expression levels in response to IFN-β as fold change (2^−ΔΔCT^); the fold change following the IFN-β treatment was significant for all genes in both monocytes and T cells (P-values<0.03, Wilcoxon signed rank test). *p-value <0.01 by Mann Whitney test for comparison of fold change between monocytes and T cells. Horizontal bars indicate median values for each column of data points.

Using the network construction function of IPA, that is based on known molecular interactions, two significant monocyte networks were selected for further evaluation, denoted as ‘cellular migration’ and ‘cellular development and proliferation’, based on suggested IPA keywords (p-value 10^−29^ for both) (Fig. 7). Matrix metalloproteinase enzyme 9 (MMP9) is the central member of figure 7A network, which also includes several other down-regulated proteases, such as cathepsin S (CTSS); cell surface proteins that are related to migration, such as CD44; and signal transduction proteins, such as NOTCH1 and MET. The second network, ‘cellular development and proliferation’, connects myelin basic protein (*MBP*) with three sub-networks which center on *IL1B*, retinoid X receptor alpha (*RXRA*) and microRNA124 (miR124) genes, which together span 70% of all network interactions (Fig. 7B). From these two networks three representative genes were validated by RT-PCR: Endothelin-1 isoform 2 (*EDN1*), which has functional relevance to immune cell adhesion and permeability of the blood-brain barrier [44], [45], *RXRA*, a nuclear hormone receptor associated with innate immune response genes [46], and Interleukin 1 beta (*IL1B*), an important mediator of the inflammatory response produced by activated macrophages. RT-PCR analysis confirmed that these genes were differentially expressed following IFN-β treatment of TNF-α activated monocytes (Fig. 7C). The IFN-β response for *EDN1* and *IL1B* was significantly smaller in magnitude in T cells compared to monocytes, demonstrating the cell-specific nature of this response.

## Discussion

In this study we identified genes with an expression pattern that is modulated by TNF-α and IFN-β in a cell-specific manner. Notably, we were able to identify a large number of genes that were differentially expressed in the TNF-α-activated and IFN-β-treated monocytes, but not, or to a significantly lesser extent, in T cells. The PBMC cell population, containing both monocytes and T cells, displayed a response that was intermediate between these two cell types. Many of the IFN-β response DEGs identified in the monocytes were genes with functions related to immune activity, such as cell migration, as expected for this cytokine's effects; however, most DEGs in the TNF-α-activated and IFN-β-treated monocytes did not cluster to known networks and canonical pathways. These results suggest the existence of a gap in the available knowledge databases with respect to biological interactions and functional links between transcripts in small subsets such as monocytes, and on the combined effect of two or more cytokines.

The present study employed a study design that evaluated the effect of IFN-β on the background of pre-exposure of the cells to TNF-α. This experimental setup was aimed to mimic in part the in vivo state, where the immune system cells are likely to encounter IFN-β while in the activated state, induced by inflammation or viral exposure, and characterized by elevated TNF-α, as reported for several immune diseases [3]. The combined activity of TNF-α and IFN-β has been reported to induce a synergistic interaction which affects gene expression patterns and activates multiple signaling pathways. Moreover, this interaction has strong antiviral effects, which are distinct from those induced by either cytokine alone [47]. Whereas the monocyte gene expression IFN-β response appeared to be strongly modulated by prior exposure to TNF-α for DEGs assessed, we did not observe a similar response in the T cells or in the PBMC mixed cell population. Since the extracellular environment is complex and is likely to contain various cytokine combinations at each given time, the in vitro assessment of single cytokine responses, fails to replicate these combinations and will miss some of the gene expression responses that may be more relevant in vivo. In this study, we did not assess the time dependency of the gene expression response to TNF-α exposure, IFN-β exposure, or both. As different sets of genes are likely to be induced at different exposure times [27], [48], further analysis of shorter or longer terms of exposures to TNF-α and IFN-β can allow in depth comprehension of endogenous and therapeutic effects of IFN-β during inflammation.

Several in vitro and ex vivo studies have utilized the whole genome approach to study the transcriptional response to IFN-β of PBMC and T cells [21], [22], [27], [49]–[51]. Many of the genes identified in the present study as IFN-β response genes in monocytes were not reported in these studies. Hence, our data, and results from other studies [52] demonstrate that gene expression of minor cell subsets within the PBMCs may remain undetected in studies employing the mixed cell population. The importance of studying minor cell subsets is further emphasized when considering the functional differences within subsets of leukocytes such as T cells and the Th1/Th2 or Treg sub-classifications, and M1 or M2 macrophages [53]. Hence, comprehension of the biological consequences of the action of different cytokines on distinct cell-subset activities requires further evaluation.

Our results highlight the HMGB1 signaling pathway as an IFN-β response pathway in monocytes. HMGB1 is a ubiquitous damage-associated molecular patterns (DAMP) cytokine, which also acts as a nucleic acid sensor by binding to immunogenic nucleic acids to initiate type I IFN signaling [54]. HMGB1, which is secreted by activated macrophages and other immune cells and released by dead cells, promotes inflammation by increasing the expression of adhesion molecules and encouraging leukocyte recruitment [55]. HMGB1 protein has been detected in the active lesions of MS [56] and elevated plasma HMGB1 levels are associated with disease activity in systemic lupus erythematosus [57] and other autoimmune diseases [58]. Because TNF-α increases the expression of HMGB1 [55], its down-regulation by IFN-β was more evident in the study design we employed. Moreover SIGLEC10– one of the differentially down-regulated transcripts we observed in monocytes – and HMGB1, have been shown to interact with each other [37]. The trimolecular complex that is formed by Siglec10, HMGB1 and CD24, the latter acting as an intermediary protein, was suggested to be part of the mechanism by which the innate immune system distinguishes between danger and pathogen associated signals (i.e. DAMP and PAMP) [37]. Thus, our results, showing that the HMGB1 and SIGLEC10 transcripts are both down-regulated, suggest that IFN-β down-regulates DAMP signaling in the innate immune system.

The pathway analyses identified a ‘cellular migration’ network (Fig. 7A) as down-regulated by IFN-β in monocytes, in line with the known effects of IFN-β on macrophage migration [59]. This network contains proteolytic enzymes, such as MMP9 and the cysteine protease cathepsin S, both reported as involved in leukocyte migration and its modulation by IFN-β [60]–[62]. RNA and protein levels of MMP9 and cathepsin S correlate with disease activity, and decrease in MS patients following IFN-β treatment [63], [64]. The gene expression changes in monocytes may thus contribute to the overall decrease in leukocyte migration that IFN-β is known to induce.

One of the DEGs we identified as highly up-regulated by the combined treatment of TNF-α and IFN-β was CD38, a pleiotropic protein, that can promote the migration of activated leukocytes, including mature dendritic cells, to the periphery and lymph nodes [65]–[67]. This gene was previously reported as up-regulated in PBMCs [27]; however, here we demonstrate a specific enhancement of the IFN-β up-regulation following TNF-α activation in monocytes. Up-regulation of CD38 by IFN-β in the pro-inflammatory environment of TNF-α activation is intriguing, since it is in apparent contradiction with the overall down-regulation of the ‘cellular migration’ network (Fig. 7A), as well as with well-established reports on down-regulation of monocyte migration by IFN-β [59], [60], [68]. Nevertheless, IFN-β therapy is known to induce, especially at the early stages of treatment of MS patients, also pro-inflammatory effects, expressed clinically by flu-like symptoms [17]. Since CD38 has many diverse activities, including functions of a receptor molecule, an ectoenzyme, and activator of second messenger pathways, further experiments are required to understand how IFN-β and CD38 interact in the context of monocyte migration. Elevated levels of CD38 and CD83, which were shown to be laterally associated on the cell membrane, are present during the maturation process of the monocytes to a dendritic cell phenotype, a process known to require IFN type 1 activity [65], [69]. Our results suggest that these two genes are both modulated by IFN-β activity in monocytes in a response distinct from that observed in T cells.

The transcripts included in the ‘cellular development and proliferation’ network (Fig. 7B) in monocytes are involved in diverse functions such as hematopoietic proliferation (IL1B, LAG3), lipid metabolism (ACADVL, CYP1B1), RNA transcription (RXRA) and ion transport (ABCC3). Therefore, the overall functional effect of IFN-β in this network is unclear. MiR124, which is one of the focal nodes, was recently reported to be a novel modulator of macrophage activation [70], [71]. Inhibition of miR-124 expression has been shown to lead to increased IL6 production and phosphorylation of STAT3, a downstream target of IL6R. Thus, miR-124 might be a regulator of the IL6-STAT3 inflammatory response [72]. The down-regulation of miR124 target genes we observed as part of the IFN-β response in monocytes indicates increased activity of miR124 following IFN-β treatment. Thus, IFN-β may act to tilt the immune activity balance towards a decrease of the IL6-STAT3 inflammatory response. Whether indeed miR124 is down-regulated by IFN-β activity, and the nature and cell specificity of the interaction of the cytokine with the miR remain to be further explored. The MBP gene is another central node in this network. In the context of monocyte cellular activity, it is likely that the down-regulated MBP transcripts encode the Golli-MBP isoforms that are known to be expressed in myeloid-lineage cells, and are up-regulated in activated macrophages [73]–[75]. Golli-MBP has been shown to have a role in T cell activation and regulation of calcium influx [73]. However, the role of golli-MBP in monocytes, and the derivative macrophages or dendritic cells, is yet to be unveiled.

IFN-β activates the transcription of more than 300 IFN response genes that harbor ISRE motifs in their promoters [13]. Accordingly, the enrichment of ISRE motifs in the promoters of T cells DEGs was anticipated. In monocytes, however, no enrichment of ISRE, or any other known motif was detected in the DEG promoters. This observation suggests that IFN-β signaling in monocytes may modulate transcription using binding sites that are different from the classical ISRE and IRF motifs. The higher prevalence of down-regulated transcripts in monocytes following IFN-β treatment is another indication for the presence of a different type of transcriptional regulation by IFN-β.

In the present study, we have shown that extensive and diverse cellular changes occur in TNF-α-activated monocytes following exposure to IFN-β. By focusing on the response of distinct cell types and by evaluating the combined effects of two cytokines with pro- and anti-inflammatory activities, we were able to identify new IFN-β response pathways and genes, some of which were cell type-specific. The modulation of expression of some of the genes by exposure to both TNF-α and IFN-β, suggests the presence of an altered functional response to IFN-β under inflammatory conditions. Analysis of the activities of other cytokines on distinct cell types of the immune system is likely to reveal additional modulatory pathways that contribute to the overall activities of the innate and adaptive immune responses.

## Materials and Methods

### Isolation of T Cells and Monocytes and Exposure to TNF-α and IFN-β

Leukocytes from six healthy volunteers between the ages of 18–65 were purchased from the Israeli Central Blood Bank. T cells were isolated by negative selection using the RosetteSep Human T cell Enrichment Cocktail according to the manufacturer's protocol (StemCell Technologies Inc., Vancouver, BC, Canada). PBMC were purified using Lymphocyte Separation Medium (MP Biomedicals, LLC, Illkirch Cedex, France) as previously described [76], and monocytes were isolated by negative selection using the EasySep Human Monocyte Enrichment kit (StemCell Technologies). The purity of the cell subsets as determined by flow cytometry (see below) was at least 90%. T cells were grown in RPMI 1640 medium containing 10% fetal calf serum, 2mM L-glutamine and 100 U/ml penicillin and streptomycin (Biological Industries, Bet-HaEmek, Israel). The monocyte medium contained, in addition to all the T cell medium components, 1% MEM non-essential amino acids solution, and 1mM sodium pyruvate (Biological Industries, Bet-HaEmek, Israel). Cells were suspended at 2*10^6^ cells/ml, incubated with recombinant human TNF-α (10ng/ml, R&D Systems, Minneapolis, MN, US) for 2 hours, and then exposed to recombinant human IFN-β1a (100 U/ml, PBL InterferonSource, Piscataway, NJ, US) for 16 hours in 5% CO_2_ at 37°. TNF-α exposure time was determined based on published time courses of total gene expression profiles [27], [48]. The 16 hours IFN-β exposure time was selected following time course assessment of the gene expression for known IFN-β response genes (described below), and evaluation of 4, 8, and 16 hours (data not shown). IFN-β concentrations were selected for compatibility with published serum levels of MS patients under IFN-β1a therapy [77]. Following treatments, cell pellets were suspended in the lysis buffer included in the HighPure RNA Isolation kit (see below) and frozen for subsequent RNA purification.

### Flow Cytometry Analysis

The purity of separated monocytes and T cells preparations was determined with anti CD3-FITC and anti CD14-PE antibodies. Staining procedures were according to the manufacturer's protocol (IQP-519F and IQP-143R respectively, IQ products, Groningen, The Netherlands), and antibody detection was performed on a FACS-CaliburR flow cytometer (Becton-Dickinson, Mountain View, CA, USA). CD38 expression experiments assessed anti-CD38-PerCP (Biolegend, San Diego, CA, US), anti CD3-FITC and anti CD14-APC (eBioScience, San Diego, CA) binding to monocyte cell surface on a CyAN ADP cytometer (Beckman Coulter, Fullertone, CA, US). Data was analyzed using the FlowJo software (Tree Star, Ashland, OR).

### RNA, cDNA Preparation and RT-PCR

RNA (HighPure RNA isolation kit, Roche Applied Science, Mannheim, Germany) and cDNA (M-MLV reverse transcriptase and random hexamer primers, Promega, Fitchburg, WI, US) were prepared according to the manufacturers' instructions. RNA quantity was measured using NanoDrop (Nanodrop Technologies, LLC, Wilmington, DE, US), and the quality was determined by the Experion system (BioRad, Hercules, CA, US). Four known IFN-β response genes were used as positive controls for IFN-β response in RT-PCR reactions, using the following primers from Applied Biosystems (Foster City, CA, US): *IFIT1* (Hs00356631_g1), *IFIT3* (Hs00155468_m1), *STAT1* (Hs00234829_m1) and *MX1* (Hs00182073_m1), as well as 4 TNF response markers, *INDO*, *ICAM1*, *IL1B* and *TRAF1* (primers sequences in Table 6). *UBE2D2* was used as a reference gene using either Taqman (HS00366152_M1) or SYBR-green RT-PCR assays (Table 6). Specific gene primers were designed by Universal Probe Library design center (Roche Applied Science), qPrimerDepot (http://primerdepot.nci.nih.gov), RTPrimerDB (http://www.rtprimerdb.org), Gene Runner (http://www.generunner.net) or BioWWW.net (http://biowww.net), synthesized by Sigma-Aldrich (St. Louis, MO) or IDT Integrated DNA Technologies (Jerusalem, Israel), and evaluated using ABsolute blue qPCR SYBR green ROX reaction mix (Abgene, Thermo Fisher Scientific, Rockford, IL, US) in RT-PCR assays. Reactions were performed in duplicates using the ABI 7300 sequence detection system, and relative quantification was calculated by the comparative C_T_ method described elsewhere, and is shown as fold change of expression (2^−ΔΔCT^) [78].

**Table 6:** Primer sequences used in this study.

Gene symbol	Entrez Gene ID	Forward	Reverse	Source
INDO	3620	GATGAAGAAGTGGGCTTTGC	TCAACTCTTTCTCGAAGCTGG	qPrimerDepot
ICAM1	3383	TGATGGGCAGTCAACAGCTA	AGGGTAAGGTTCTTGCCCAC	qPrimerDepot
IL1B	3553	GAAGCTGATGGCCCTAAACA	AAGCCCTTGCTGTAGTGGTG	qPrimerDepot
TRAF1	7185	CTATAAGCCCAGGAAGCCG	CTTCCCTTGAAGGAGCAGC	qPrimerDepot
ASCL2	430	TCTAAGGGCACAGAGAATCCAT	ACAAAAATAGGTCTCCGTTTGC	UPL^1^
CD38	952	CAGACCGTACCTTGCAACAA	AGGTCATCAGCAAGGTAGCC	UPL
CD83	9308	CTCCAGCTTCTGCTCCTGA	GCAAGCCACCTTCACCTC	UPL
LTA4H	4048	TCACGGTCCAGTCTCAGGA	TTTTCTATTGTAAGGTCCTTTGTATCC	UPL
RIPK2	8767	TCCACTCTCAACTGCAGGAA	GGATCCACTGCTGGGCTA	UPL
SIGLEC10	89790	TCCTATGCGGAGATGCTACTG	CAGAATCTCCCATCCATAGCC	UPL
TBXAS1	6916	CACTTTCTTTTGCCACCTACCT	TTCCTCGAGGCTGCAGAA	UPL
TRIM25	7706	TCCGAACTCAACATCTCTCAAG	GCGGTGTTGTAGTCCAGGAT	UPL
UBE2D2	7322	ATTGAATGATCTGGCACGGG	GTCATTTGGCCCCATTATTG	RTPrimerDB
IL1A	3552	ACTGCCCAAGATGAAGACCA	CCGTGAGTTTCCCAGAAGAA	qPrimerDepot
HMGB1	3146	CCCAAAAGCGTGAGCTTAAAAT	AGTCTCGTTTCCTGAGCAGTCC	Yang et al. [80]
IL8	3576	GCCAAGGAGTGCTAAAGAAC	TTCTCCACAACCCTCTGCAC	Gene Runner
EDN1	1906	TATCAGCAGTTAGTGAGAGG	CGAAGGTCTGTCACCAATGTGC	Biowww.net
RXRA	6256	TTCGCTAAGCTCTTGCTC	ATAAGGAAGGTGTCAATGGG	Biowww.net

1 UPL – Universal Probe Library.

### Gene Expression Analysis

All RNA samples for gene expression analysis had a RNA Integrity Number (RIN) value above 7.5 using the Experion system. Gene expression microarray analysis was performed on monocytes and T cells from three donors, using the Illumina Human-6 v3 BeadChips, (Illumina, San Diego, CA). The raw data was deposited in a MIAME compliant database (GEO, accession number GSE34627). Each chip contained ∼47,000 probes derived from the National Center for Biotechnology Information Reference Sequence (NCBI) RefSeq Release 37 and other sources. The microarray experimental procedures (from cDNA synthesis to raw data normalization) were performed by the Genomics Core Facility at the Rappaport Faculty of Medicine & Research Institute in the Technion according to the manufacturer's instructions. The intensity of the bead fluorescence was detected by the Illumina BeadArray Reader, and the raw data analyzed using BeadStudio v3.4 software.

### Statistical Analysis

The raw gene expression data was exported from BeadStudio and imported into JMP Genomics V5 software (SAS Institute Inc, Cary, NC). Quality control and analysis in JMP Genomics was done on log2 transformed data, after filtering for non-expressed genes (detection p-value<0.01), and for low variance transcripts across samples (variance <5%). Using batch normalization, the gene expression data set was corrected for difference of variation between chips, but no additional normalization was required. The data was analyzed using 2-way ANOVA for cell type (T cells or monocytes) and IFN-β exposure (yes or no), as well as the interaction between these two factors (cell-type*IFN-β). DEGs were defined as transcripts that had a fold change of expression ≥2 and a corrected p-value ≤0.05 using the False Discovery Rate (FDR) [23]. In addition, each simple paired combination, i.e. either monocytes or T cells, with and without IFN-β was also compared using t-test, and DEGs were defined as previously described. A hierarchical clustering of the 50 top DEGs within each cell type was performed using JMP Genomics. Volcano plots were generated by the R Project for Statistical Computing version 2.12.1 [79]. The Mann Whitney test was used to compare RNA levels between cell subsets. Significance of fold change following IFN-β exposure for RNA levels was tested by the Wilcoxon signed rank test.

### Functional Pathways Analyses

Evaluation of enriched biological functions was performed using GOrilla (http://cbl-gorilla.cs.technion.ac.il) [40]. Data were imported into GOrilla as transcript lists ranked by adjusted p-values, as obtained in the simple paired comparisons and by the fold change for each cell type. The gene lists were analyzed using the SPIKE program, for detection of enriched cell signaling pathways, and PRIMA for identification of enrichment of transcription factor binding sites in the promoters of the DEG list, as implemented in the Expander v5 analysis program [41], [42].

The Ingenuity Pathway Analysis (IPA 8.0, Ingenuity® Systems, Redwood City, CA, US) was used to identify statistically significant functional categories in the data set. Simple paired comparisons lists were imported into IPA and filtered using p-value = 0.05 and fold change = 1.3 as a cutoff. Up- and down-regulated genes were both included in the core analysis. DEGs were mapped to canonical pathways and tested by the Fisher's Exact Test p-value which represents the probability to find the same number of DEGs in a pathway of the same size composed of randomly selected genes. Genes were mapped into IPA-generated networks based on their order of connectivity. P-values were calculated for each network using the Fisher's exact test. Networks with p-value ≤10^−25^ were considered to be significantly enriched.

## Supporting Information

Figure S1 Workflow of the experimental procedures and subsequent gene array analyses for differential expression.
(TIF)
