# Supplementary figures and images for: Interferon-Beta Induces Distinct Gene Expression Response Patterns in Human Monocytes versus T cells
Source: PLoS One. 2013 Apr 23;8(4):e62366. doi: 10.1371/journal.pone.0062366 (PMC3633862; doi:10.1371/journal.pone.0062366)

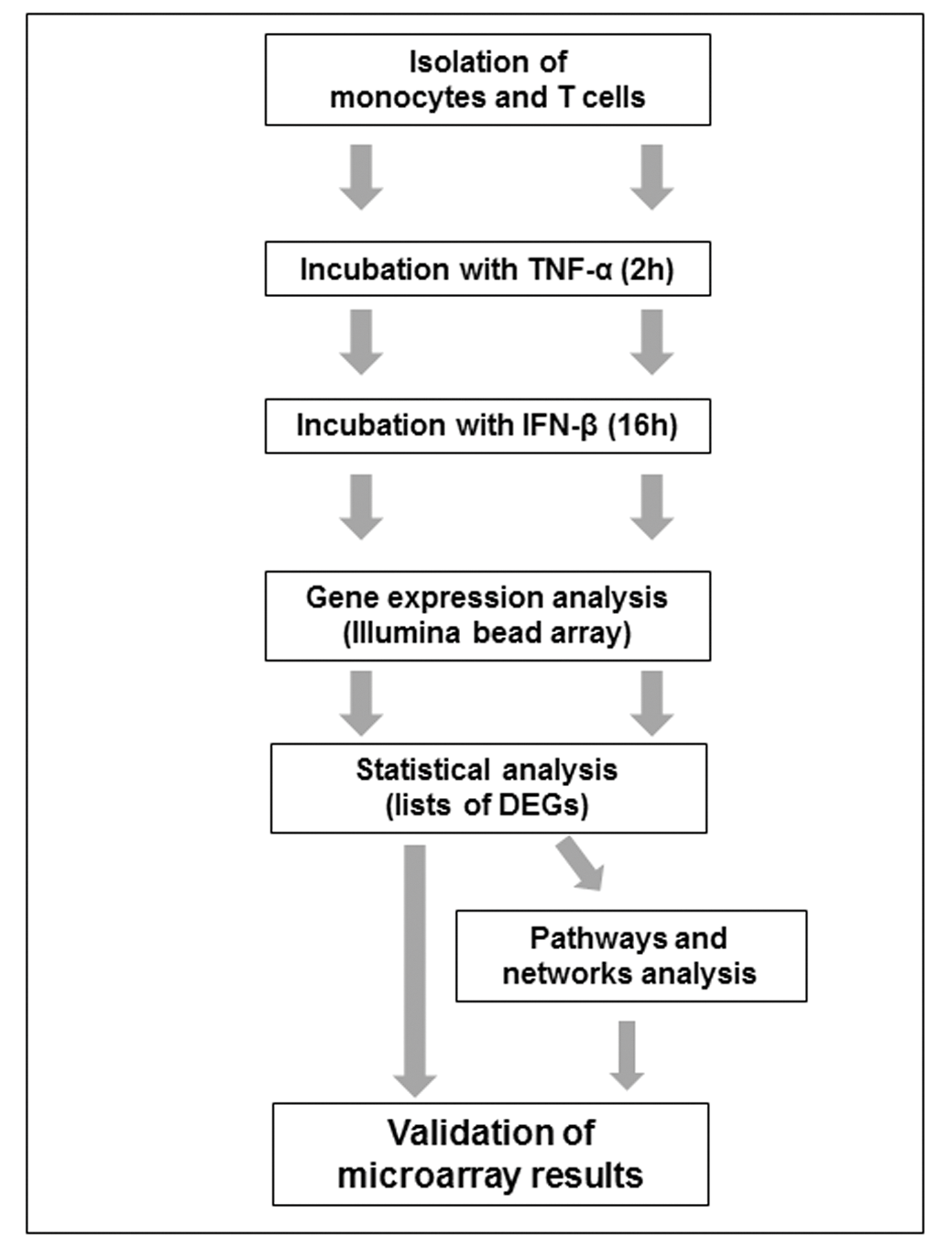

Supplement: Figure S1 — Workflow of the experimental procedures and subsequent gene array analyses for differential expression. (TIF) [file pone.0062366.s001.tif]
